# RETRACTED: Zhang, E. Hepatic PLIN5 Deficiency Impairs Lipogenesis through Mitochondrial Dysfunction. *Int. J. Mol. Sci.* 2022, *23*, 15598

**DOI:** 10.3390/ijms242015458

**Published:** 2023-10-23

**Authors:** Enxiang Zhang

**Affiliations:** 1 Key Laboratory of Growth Regulation and Transformation Research of Zhejiang Province, School of Life Sciences, Westlake Institute for Advanced Study, Westlake University, Hangzhou 310024, China; enxiang.zhang@duke.edu or zhangenxiang0728@163.com; 2 Department of Cell Biology, Duke University Medical Center, Durham, NC 27710, USA; 3 Duke Regeneration Center, Duke University Medical Center, Durham, NC 27710, USA

The *International Journal of Molecular Sciences* retracts the article entitled “Hepatic PLIN5 Deficiency Impairs Lipogenesis through Mitochondrial Dysfunction” [1].

Following publication, the author contacted the Editorial Office with concerns regarding the ownership of the data presented in this manuscript.

Adhering to our complaints procedure, an investigation was conducted that confirmed that appropriate permission had not been gained to publish the data contained in the article [1]. This lack of appropriate permission was confirmed by the University of Minnesota, and the article is therefore retracted. 

This retraction was approved by the Editor in Chief of the *Int. J. Mol. Sci*. 

The author agreed to this retraction. 

## References

[1] Zhang E. (2022). RETRACTED: Hepatic PLIN5 Deficiency Impairs Lipogenesis through Mitochondrial Dysfunction. Int. J. Mol. Sci..

